# Brain functional connectivity and local coherence in non-converters with clinical high risk for psychosis

**DOI:** 10.1192/j.eurpsy.2023.1277

**Published:** 2023-07-19

**Authors:** Y. Panikratova, E. Abdullina, I. Lebedeva, M. Omelchenko, D. Tikhonov, V. Kaleda

**Affiliations:** Mental Health Research Center, Moscow, Russian Federation

## Abstract

**Introduction:**

Investigation of resilience mechanisms in patients with clinical high risk for psychosis (CHR) may inform clinical practice for the development of early intervention programs. Resilience mechanisms in CHR who did not transit to psychosis for a long period of observation may be more pronounced than in CHR converters.

**Objectives:**

We aimed to compare CHR who did not convert to psychosis for 7.3 ± 1.7 years, patients with first-episode psychosis (FEP), and healthy controls (HC) in terms of brain functional connectivity and local coherence.

**Methods:**

Twenty-seven CHR (mean age 27.5 ± 3.1), 24 FEP (mean age 20.6 ± 3.6), and 27 HC (mean age 27.3 ± 4) underwent resting-state fMRI (3T). All participants were males. Functional connectivity between 32 regions of interest (components of default mode, sensorimotor, visual, salience, dorsal attention, frontoparietal, language, and cerebellar networks; CONN functional network atlas www.nitrc.org/projects/conn) and whole-brain local coherence (LCOR; Deshpande et al. HBM 2009; 30(1) 13-23) were compared between 3 groups of participants (one-way ANCOVA) with *post hoc* analyses.

**Results:**

CHR and HC demonstrated higher functional connectivity between the occipital cortex (visual network) and right rostral prefrontal cortex (salience network) compared to FEP. CHR also showed higher local coherence in the right calcarine and cuneal cortex than FEP (the following differences did not survive the correction for multiple comparisons: CHR>HC and HC>FEP).

**Conclusions:**

Our findings on brain functional connectivity and local coherence may be considered as the markers of resilience mechanisms in patients with CHR as these parameters were different between CHR and FEP and were similar in CHR and HC.

**The research was supported by RFBR grant project 20-013-00748.**

**Disclosure of Interest:**

None Declared

